# The Holy Grail of Orthopedic Surgery: Mesenchymal Stem Cells—Their Current Uses and Potential Applications

**DOI:** 10.1155/2017/2638305

**Published:** 2017-06-18

**Authors:** Roberto Berebichez-Fridman, Ricardo Gómez-García, Julio Granados-Montiel, Enrique Berebichez-Fastlicht, Anell Olivos-Meza, Julio Granados, Cristina Velasquillo, Clemente Ibarra

**Affiliations:** ^1^Tissue Engineering, Cell Therapy and Regenerative Medicine Unit, Instituto Nacional de Rehabilitación “Luis Guillermo Ibarra Ibarra”, Calzada México Xochimilco, No. 289, Col. Arenal de Guadalupe, 14389 Mexico City, Mexico; ^2^School of Medicine, Faculty of Health Sciences, Anáhuac University, Av. Universidad Anáhuac 46, Lomas Anahuac, 52786 Huixquilucan, MEX, Mexico; ^3^Department of Orthopedic Surgery, American British Cowdray Medical Center, Av. Carlos Graef Fernández, No. 154, Col. Santa Fe, Cuajimalpa, 05300 Mexico City, Mexico; ^4^Orthopedic Sports Medicine and Arthroscopy Division, Instituto Nacional de Rehabilitación “Luis Guillermo Ibarra Ibarra”, Calzada México Xochimilco, No. 289, Col. Arenal de Guadalupe, 14389 Mexico City, Mexico; ^5^Department of Transplantation, Instituto Nacional de Ciencias Médicas y Nutrición “Salvador Zubirán”, Av. Vasco de Quiroga, No. 15, Tlalpan, Belisario Domínguez Sección XVI, 14080 Mexico City, Mexico; ^6^Direction of Research, Instituto Nacional de Rehabilitación “Luis Guillermo Ibarra Ibarra”, Calzada México Xochimilco, No. 289, Col. Arenal de Guadalupe, 14389 Mexico City, Mexico; ^7^General Direction, Instituto Nacional de Rehabilitación “Luis Guillermo Ibarra Ibarra”, Calzada México Xochimilco, No. 289, Col. Arenal de Guadalupe, 14389 Mexico City, Mexico

## Abstract

Only select tissues and organs are able to spontaneously regenerate after disease or trauma, and this regenerative capacity diminishes over time. Human stem cell research explores therapeutic regenerative approaches to treat various conditions. Mesenchymal stem cells (MSCs) are derived from adult stem cells; they are multipotent and exert anti-inflammatory and immunomodulatory effects. They can differentiate into multiple cell types of the mesenchyme, for example, endothelial cells, osteoblasts, chondrocytes, fibroblasts, tenocytes, vascular smooth muscle cells, and sarcomere muscular cells. MSCs are easily obtained and can be cultivated and expanded in vitro; thus, they represent a promising and encouraging treatment approach in orthopedic surgery. Here, we review the application of MSCs to various orthopedic conditions, namely, orthopedic trauma; muscle injury; articular cartilage defects and osteoarthritis; meniscal injuries; bone disease; nerve, tendon, and ligament injuries; spinal cord injuries; intervertebral disc problems; pediatrics; and rotator cuff repair. The use of MSCs in orthopedics may transition the practice in the field from predominately surgical replacement and reconstruction to bioregeneration and prevention. However, additional research is necessary to explore the safety and effectiveness of MSC treatment in orthopedics, as well as applications in other medical specialties.

## 1. Introduction

Very few tissues and organs can spontaneously regenerate following disease or trauma, and this regenerative capacity diminishes during the lifetime. As such, scientists are developing techniques in the fields of tissue engineering, cell therapy, and regenerative medicine to aid the regeneration of the musculoskeletal system. Stem cell (SC) use in orthopedic surgery has the potential to change the field of orthopedics from one dominated by surgical replacement and reconstruction to one of bioregeneration and prevention [1].

Around the 1960s, a unique group of bone marrow cells was discovered with the capability to differentiate into various other cells [2, 3]. However, we now know that several types of SCs exist, each with different characteristics—including embryonic stem cells (ESCs), fetal stem cells (FSCs), infant stem cells, and adult stem cells, from which mesenchymal stem cells (MSCs) derive [4]. Adult and fetal SCs are considered to be undifferentiated; they can be found in adult tissues and in the fetus, respectively [4]. Various legal, ethical, physiological, and immunologic concerns are associated with the use of ESCs and FSCs, which have limited their application [5]. Nevertheless, most medical specialties can benefit from the progress in SC research and regenerative medicine. More than 3000 trials regarding SC research in musculoskeletal diseases are currently underway [5]. There are multiple clinical opportunities for SCs in orthopedic surgery, ranging from tissue regeneration and modulation of the immune function, to the modeling of rare diseases [5].

**Figure 1:**
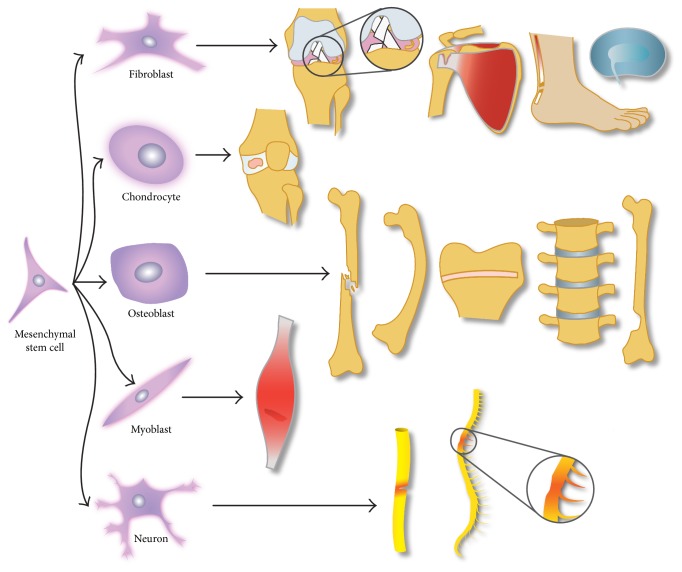
Mesenchymal stem cells (MSCs) are broadly applicable to the field of orthopedics. MSCs can be stimulated to differentiate into several cellular lineages with various clinical applications. For example, fibroblasts can be used to regenerate torn or injured tendons, ligaments, menisci, rotator cuff, and intervertebral disc; chondrocytes can be used to regenerate articular cartilage defects and treat osteoarthritis; osteoblasts can facilitate fracture consolidation and treat metabolic bone diseases such as osteogenesis imperfecta, growth cartilage diseases, spinal fusion, and regeneration of segmental defects of the bone after tumor removal; myoblasts can be used to regenerate torn or injured muscles; and neurons can be used to regenerate peripheral nerves or aid in the repair of traumatic spinal cord injuries.

MSCs can be obtained from the umbilical cord, amniotic fluid, placenta, adipose tissue, joint synovium, synovial fluid, dental pulp, endosteum, and periosteum [2–4, 6, 7]. Steinert et al. [8] have also recently identified MSCs in the bursa subacromialis in adults [8]. One theory for the varied locations of SCs is that these cells derive from pericytes [9–11]. Moreover, MSCs are multipotent, meaning they can differentiate into multiple mesenchymal cell types—including endothelial cells, osteoblasts, chondrocytes, fibroblasts, tenocytes, vascular smooth muscle cells, myoblasts, and neurons (Figure 1). Recent publications report that MSCs can also differentiate into nonmesodermal cells—such as neurons, astrocytes, and hepatocytes—in vitro [3, 12, 13]. Further, by being reservoirs of repair cells, exerting immunomodulatory and anti-inflammatory effects, endogenous MSCs contribute to the preservation of healthy tissues [14].

**Figure 2:**
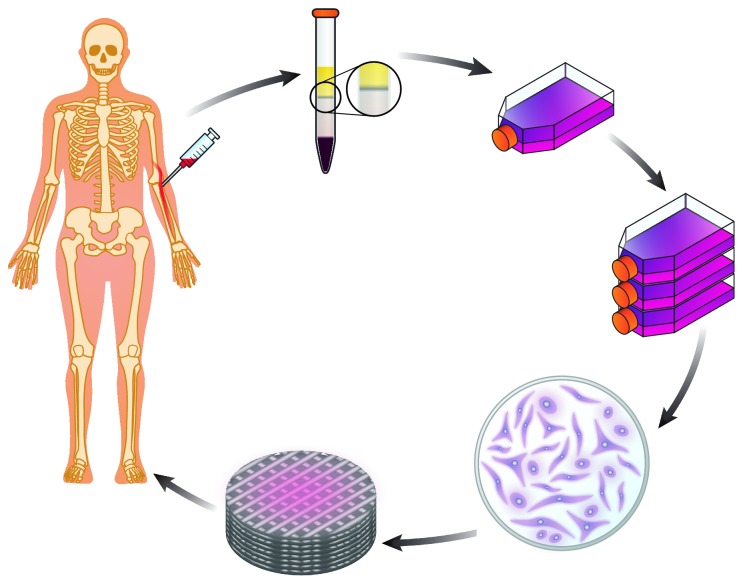
MSC isolation procedure. Blood from a peripheral vein is collected from the patient following the mobilization of MSCs to the peripheral circulation with granulocyte colony-stimulating factor. Mononuclear cells are then separated from other blood components using a density gradient and seeded in cell culture flasks where they will proliferate. Expanded cells can then be stimulated to differentiate into a particular cellular lineage, harvested, and seeded onto a biocompatible scaffold for implantation in the patient, as required.

As already mentioned, MSCs can be obtained from virtually any tissue in the body. For regenerative medicine and tissue engineering purposes, MSCs are usually obtained from the bone marrow, which has an MSC content of approximately 1 : 10^4^ to 1 : 10^5^ bone marrow mononuclear cells [2, 6, 7]. The prevalence of MSCs in the peripheral circulation is much lower, around 1 : 10^8^ peripheral blood mononuclear cells [2, 6, 7]. Obtaining the bone marrow aspirate is an invasive procedure that regularly necessitates general anesthesia and can be associated with pain, discomfort, and complications [6, 7]. Therefore, SC research has focused on identifying agents that promote MSC egress from the bone marrow into the peripheral circulation to facilitate their obtention and isolation. The most widely used agent is the granulocyte colony-stimulating factor (G-CSF) filgrastim, which is usually given by subcutaneous injection in conjunction with chemotherapy in hematological cancer patients [13, 15]. Filgrastim acts by disrupting the interaction involving vascular cell adhesion molecule-1 (VCAM-1) in the vascular stroma and integrin *α*4*β*1 (also known as very late antigen-4 [VLA-4]) in the cells. Consequently, MSCs are mobilized from the bone marrow stroma to the peripheral circulation [9, 12, 13, 15]. The blood is then obtained from the peripheral circulation, and MSCs are isolated, expanded, differentiated, and seeded on scaffolds (Figure 2).

In 2006, the International Society for Cellular Therapy recommended that cells should fulfill the following criteria to be considered as MSCs: (1) the cells must be plastic-adherent when maintained under standard culture conditions; (2) they must express CD73, CD90, and CD105 markers and should not express CD34, CD45, CD14, HLA-DR, CD11b, or CD19; and (3) they should be able to differentiate into osteoblasts, chondroblasts, and adipocytes in vitro [16].

MSCs possess immunomodulatory and immunosuppressive properties via the secretion of specific cytokines and can thus modulate inflammation following an injury [13]. MSCs are hypoimmunogenic and can evade the host immune system. This is in part because MSCs express less major histocompatibility complex (MHC) class I molecules, which avoids MSCs from removal by natural killer cells. Also, MSCs do not express MHC class II molecules on their cell surface, which in turn gives the potential to avoid identification by alloreactive CD4^+^ T cells [13, 22]. MSCs have the ability to interact with immune cells and can suppress and modulate alloreactivity. Further, MSCs can hamper T cell proliferation and activation by secreting soluble factors like hepatocyte growth factor, TGF-*β*1, IL-10, and prostaglandin E2 [13, 22]. Nevertheless, these immunomodulatory and immunosuppressive properties have not been completely established in orthopedic applications.

One of the issues related with regenerative cell-based therapies is the risk of tumor formation. However, in 2013, Hernigou and colleagues completed a 12.5-year follow-up of 1873 patients receiving autologous bone marrow-derived stromal progenitors, but found no increase in cancer risk at treatment sites or other untreated areas based on imaging studies [17]. This suggests that the application of these cells is somewhat safe and it does not increase the risk of tumor formation.

This review will discuss the major current and potential future applications of MSCs in orthopedic surgery.

## 2. MSCs and Orthopedic Trauma

The endosteum and periosteum are rich sources of osteochondral progenitor cells during fracture healing [18]. Grafting experiments revealed that the transplanted periosteum generates both osteoblasts and chondrocytes during fracture repair, whereas the endosteum generates primarily osteoblasts [18]. In addition, bone morphogenetic protein (BMP)-2 has been shown to stimulate chondrogenesis within the periosteum, but not in the endosteum, indicating that cells within these sites may be activated by different factors [13, 18].

Fracture healing is an intricate process that occurs through a combination of endochondral and intramembranous ossification [19]. During endochondral ossification mechanism, a cartilage template is initially formed and subsequently replaced by osteoblasts delivered to the fracture site as a result of angiogenesis [19]. Recent work has also shown that hypertrophic chondrocytes in the fracture callus may persist and transdifferentiate into osteoblasts [20, 21]. Thus, the potential application of these mechanisms in bone healing treatments, for example, therapeutic stimulation of the conversion of chondrocytes into osteoblasts in cases of hypertrophic nonunion should be researched further [20, 21].

Cells that contribute to the healing of a fracture can be mobilized from the circulation [13]. Nevertheless, research suggests that these circulating cells account for only a small number of cells in the fracture callus under normal circumstances, suggesting that the majority of the cells at the fracture site migrated from the adjacent tissues [13]. As such, therapeutic amplification of circulating MSCs through their mobilization could also represent a potential therapeutic opportunity in fracture repair [13].

MSCs also comprise one therapeutic opportunity in such fracture complications as delayed union or nonunion. Some authors, for example, Hernigou et al. [21], used bone marrow aspirates from the iliac crest to treat atrophic diaphyseal nonunions. The aspirates were implanted exactly at the site of nonunion, leading to callus formation. Although promising, the effective dose of cells required for a successful treatment remains to be demonstrated [13, 21].

## 3. The Potential Use of MSCs in Muscle Recovery

Following skeletal muscle injury, complete functional recovery remains challenging and this recovery is delayed by the development of scar tissue. The regenerative capacity of skeletal muscle is low and mainly brought about by mononucleated precursor cells (satellite cells) [1]. Satellite cells are situated under the basal lamina that envelops every myofiber and exhibit SC-like characteristics during the repair of muscle injury [23–25]. Accordingly, the use of satellite cells represents a very appealing strategy to treat muscle disorders and injuries because of their intrinsic myogenic potential [23–25]. However, in vitro expansion of these cells is difficult as they rapidly senesce and display poor posttransplantation survival [22–25].

Bone marrow-derived mesenchymal SCs (BMDMSCs) have the ability to differentiate and blend with myoblast*s* in vitro and contribute to the healing process of the muscle [24, 25]. Adipose-derived mesenchymal SCs (ADMSCs) share several characteristics with BMDMSCs, such as the expression of specific cell surface proteins (e.g., CD90 and CD29) and the capacity to go through differentiation along the classical mesenchymal lineages [24]. Cui et al. demonstrated that ADMSCs are not as immunogenic and immunosuppressive than BMDMSCs [1, 22]. In fact, ADMSCs have several advantages over BMDMSCs [24]. For example, they are easily accessible, more abundant and proliferative, and secrete several angiogenic and antiapoptotic cytokines that sustain tissue regeneration and reduce harm. Further, Peçanha et al. [24] investigated whether allogeneic ADMSCs contributed to the healing of the skeletal muscle. They injected ADMSCs from healthy rats into the soleus muscle of rats. Tetanic muscle force was evaluated 2 and 4 weeks after the injection, and histological examination was performed to establish the deposition of collagen in the muscle and the number of centronucleated muscle fibers. The authors showed that the tetanus force and the amount of centronucleated myofibers were superior in the treated group in comparison with the control group [23, 24]. They concluded that muscle repair and force were enhanced by ADMSC therapy 2 weeks after the treatment, suggestive that ADMSC administration could indeed accelerate muscle repair [22].

Muscle-derived MSCs (MDMSCs) also play a key role in muscle healing. Upon implantation into the skeletal muscle, MDMSCs show longer-term survival than myoblasts and directly cooperate in the regeneration of myofibers [25]. Studies suggest that MDMSCs are also able to differentiate into endothelial and neural lineages in vivo, which may improve the neural and vascular supply to the regenerating muscle [25, 26].

Evidence from recent studies suggests that inflammatory pathways and signaling may affect muscle healing [26]. Numerous anti-inflammatory agents are employed following an injury, including nonsteroidal anti-inflammatory drugs (NSAIDs) that are typically prescribed for pain control. Their use may result in the upregulation of transforming growth factor (TGF)-*β*1 levels; it has been proposed that TGF-*β*1 and other cytokines play a key function in muscle healing [23, 25]. However, several other studies suggest that these drugs, also cyclooxygenase-2 inhibitors, augment fibrosis, inhibit myogenic precursor cells, and impair myofiber regeneration by specifically upregulating the synthesis of TGF-*β*1 [26]_._ These contradictory findings indicate that prudence should be exercised concerning the use of NSAIDs and cyclooxygenase-2 inhibitors after injury [26].

## 4. MSCs in Articular Cartilage Lesions and Osteoarthritis

### 4.1. MSC Therapy in the Treatment of Articular Cartilage Lesions

Articular cartilage lesions are one of the most frequent problems encountered by orthopedic surgeons [27]. Due to the relative acellularity and the specific biochemical properties of the cartilage, the self-renewal potential of this tissue is very limited [27, 28]. An orthopedist may use numerous techniques to treat articular cartilage lesions; however, none of the currently available surgical treatments for cartilage repair provides a tissue with the biomechanical and biochemical properties of native cartilage [4, 27, 28].

Microfractures—and a more recently developed nanofracture technique—are often employed to treat articular cartilage lesions. The aim of these bone-marrow stimulation techniques is to perform small perforations in the subchondral bone, releasing SCs from the bone marrow [4]. Unfortunately, the created neotissue is fibrocartilage, which differs in biomechanical and biochemical characteristics from that of the hyaline cartilage. The durability of the repaired tissue is also lower than that of native cartilage [26, 27]. More recent techniques aim to regenerate the cartilage, such as autologous chondrocyte implantation (ACI) [28, 29], using various cell types—including SCs, chondrocytes, SCs with periosteum, chondrocyte precursors, or a combination of these [4]. A recent laboratory study confirmed that autologous chondroprogenitor cells were more effective in healing articular cartilage defects as compared to allogenic cells [4]. Using a bovine model, Zhou et al. [30] found that the chondrogenic progenitor cells surrounding the superficial zone of articular cartilage more closely resemble synoviocytes and synovial fluid-derived cells than chondrocytes. These chondrogenic progenitor cells showed a predisposition to overexpress chemokines that encouraged chemotaxis of immune cells, suggestive that they interfere with inflammation after cartilage injury [30–32]. Moreover, transplantation of synovial-MSCs (SMSCs) in rabbits resulted in abundant cartilage matrix development at defect sites as described by Koga et al. [33] Interestingly, these authors also observed that SMSCs differentiated into osteocytes deeper into the defect, but differentiated into chondrocytes in the superficial zone. This investigation supports the multilineage differentiation potential of SMSCs in vivo depending on the local microenvironments [32, 33]. Furthermore, SMSCs were shown to promote cartilage regeneration upon transplantation into a full-thickness articular cartilage defect in a porcine model as early as 3 months subsequent to the procedure, evaluated by magnetic resonance, arthroscopic, and histologic examination [34]. In an equine model, the application of an autologous platelet-enriched fibrin scaffold to a full-thickness chondral defect of the knee resulted in repair of the cartilage defect, as evidenced by arthroscopy, magnetic resonance imaging T2 mapping, histology, biomechanical testing, and microcomputed tomography [35]. Also using an equine model, Frisbie et al. [36] compared the effect of various treatments (fibrin alone versus autologous chondroprogenitor cells with fibrin versus allogenic chondrogenitor cells with fibrin) on the repair of generated cartilage defects in the medial trochlear ridge of the femur. Arthroscopic, imaging and microscopy analyses after a 12-month follow-up period revealed that tissue repair was significantly more advanced in horses treated with autologous cells and fibrin than in the two other treatment groups [36].

Interestingly, in humans, the quantity of SMSCs in the synovial fluid seems to augment in the knees with osteoarthritis, degenerated cartilage, meniscus damage, and subsequent to intra-articular ligament injury [37], raising the inquiry if the amount of SMSCs mobilized from the synovium to the synovial fluid raises proportionally to the extent of cartilage degeneration as an element of the reparative mechanism [32, 37]. The human infrapatellar fat pad is a rich source of MSCs that can be easily harvested during arthroscopic procedures [38]. Infrapatellar fat pad-derived MSCs isolated from osteoarthritic patients are highly clonogenic and their chondrogenesis is similar to that of cells isolated from healthy articular cartilage [38].

Recent studies report that the biological characteristics of peripheral blood-derived MSCs and human umbilical cord blood-derived MSCs are comparable with BMDMSCs with respect to their ability to repair cartilage defects [38, 39]. The effect of platelet-rich plasma (PRP) on articular chondrocytes has been recently investigated [31]. Interestingly, PRP—containing a relatively low number of platelets and very few leukocytes—stimulates chondrocyte anabolism, as demonstrated by changes in the expression of type II collagen and aggrecan [31]. On the other hand, PRP with a high number of both platelets and leukocytes promotes catabolic chondrocyte pathways. Furthermore, Sakata et al. [40] found that PRP stimulates the secretion of superficial zone protein, a lubricant found in the articular cartilage.

### 4.2. MSC Therapy in the Treatment of Osteoarthritis

Osteoarthritis profoundly impacts the quality of life and is related with enormous social and economic costs. The beginning of degenerative changes in the joint is associated with an abnormal activity or diminution of cell reservoirs. This leads to the failure of chondrogenic potential and the prevalence of a fibrogenic chondrocyte phenotype [41]. Several clinical trials are currently investigating the delivery of MSCs to the knee via an intra-articular injection with the goal of exploiting their anti-inflammatory and immunosuppressive properties for the management of osteoarthritis and rheumatoid arthritis [41, 42]. Studies have demonstrated promising outcomes with this procedure. However, the best dose and vehicle of administration have not been well established [41]. No accepted biological therapy, pharmacological intervention, or practice is at present capable to stop the constant joint destruction in osteoarthritis. The current treatments yield only symptomatic rather than regenerative outcomes. None of these compounds exerts a clinically significant therapeutic effect to counteract the progressive loss of cartilage, which ultimately leads to joint destruction [14].

Many experiments on the beneficial effects of MSCs engross the treatment of osteochondral or chondral defects in animal models. These experiments usually utilize scaffolds with habitually contradictory results. Also, these experiments are targeted for focal acute lesions rather than the large and chronic lesions comparable to those encountered in osteoarthritis [41, 42]. Murphy et al. [43] described an outstanding therapy for posttraumatic osteoarthritis in goats. In that animal model, a medial meniscectomy and the resection of the anterior cruciate ligament (ACL) caused advanced osteoarthritis of the knee. Afterward, the injection of an intra-articular suspension of autologous MSCs was seen to evoke a meniscal repair response that resulted in clinical improvement with evidence of chondroprotection. The cells that were implanted were not identified at the cartilage, but at the surface of the regenerated meniscus. This experiment proposed that transplanted MSCs control host cell behavior via paracrine effects and operate as building blocks for the creation of repair tissue. In addition, these cells express some specific genes, such as parathyroid hormone-like hormone (*PTLH*), Indian hedgehog (*IHH*), and *BMP2*, which results in an augmented expression of type II collagen [43].

The usefulness of intra-articular delivery of MSCs to the knee has been evaluated in various preclinical models, such as rabbits, mice, rats, guinea pigs, dogs, horses and sheep [41]. In these models, MSC application inhibited osteoarthritis progression and decreased prostaglandin E_2_ levels in the synovial fluid [44]. The majority of the techniques engross the administration of MSCs via an intra-articular injection into the synovial fluid compartment by means of a scaffold-free method, generally with hyaluronan as a vehicle [44].

Articular repair has been developed through various cell-mediated techniques, a number of which targets the employment of endogenous cell populations as an alternative to the delivery of ex vivo preparations. Lee et al. [45] designed a biological scaffold covered with TGF-*β*3 that facilitated the formation of a functional and structural articular cartilage layer. In this experiment, matrix buildup and type II collagen synthesis were superior, while cellularity of the articular layer was augmented nearly threefold compared to that of the control [45].

Yet another new therapeutic approach involves the administration of the small heterocyclic molecule kartogenin, which stimulates differentiation of MSCs to a chondral lineage and helps with cartilage repair in surgery-induced and collagenase-induced models of osteoarthritis in mice [48]. Kartogenin administration was also shown to augment the cartilage thickness and improve matrix structure and weight-bearing capability, apparently by increasing chondrogenic initiation of progenitor cells in the cartilage [46–50].

## 5. MSCs and the Meniscus

The meniscus possesses distinctive reparation potential depending on the lesion location. Tears localized in the inner third of the meniscus, which is avascular, have restricted or no potential for regeneration in the absence of bleeding [51–54]. As with other orthopedic injuries, several approaches exist to treat meniscal tears. Meniscectomy is a commonly used technique but there is evidence with the succeeding development of osteoarthritis [51–53]. Meniscal sutures are also frequently used; however, the healing capacity of the tear is limited.

Research into meniscus biology has focused on the utilization of cell-based approaches to improve meniscal healing and on the use of scaffold materials to stimulate meniscal tissue regeneration [31, 51, 53, 54]. Lately, cell-based therapy for meniscal lesions has been pursued. The investigated cell sources include BMDMSCs, the synovium, vascular endothelium, and intrinsic meniscal SCs [31, 51, 53, 54]. It has been proven that chondrocytes placed onto meniscal matrices can join separate fragments of the fibrocartilage strips [51]. Over time, the histological and biomechanical properties of the meniscus, particularly the adhesion strength, increase because new cartilaginous matrix is formed [51, 53, 54]. Dutton et al. [52] showed that the application of SCs to the avascular section of the meniscus facilitates meniscus healing in the porcine model.

Studies in rabbits demonstrate that the placement of scaffolds with cultured autologous BMDMSCs facilitates meniscus regeneration [51, 53, 54]. In punch defect and menisectomy models in rabbits, the utilization of allogenic synovial MSCs has been studied [55]. A direct intra-articular administration of cells suspended in a phosphate-buffered saline led to a significant improvement in tissue regeneration at weeks 2, 4, and 8 posttreatment [53–55]. Larger defects usually call for the employment of an SC-loaded scaffold; on the other hand, smaller defects or scenarios involving augmentation of repair of the meniscus might not call for scaffold use because SCs seem to be capable to concentrate and stay at the site of repair [53, 54, 56].

## 6. MSCs for the Treatment of Bone Pathologies

Pathological conditions, such as tumors, pseudarthrosis (congenital or acquired), bone cysts, revision arthroplasty, or infections, may lead to major bone loss requiring the use of bone substitutes to re-establish the structural integrity [57, 58]. The utilization of autologous and allogenic bone grafts is frequently associated with donor site morbidity and a risk of transmission of infections [57, 58]. The purification and expansion of bone marrow cells from rats, mice, dogs, and humans have been reported, and their capability to generate bone when they are implanted ectopically with hydroxyapatite or another proper scaffold was demonstrated [57, 58]. Techniques have also been refined for the expansion of bone marrow osteoprogenitors [58], indicating the likelihood of applying autologous human stromal progenitors to treat bone defects.

Osteoproduction, osteoinduction, osteoconduction, and mechanical stimulation are hallmarks of successful bone tissue engineering [59, 60]. Osteoproduction indicates the ability of the cell to secrete bone matrix. Researchers have succeeded in healing critically sized bone defects with purified BMDMSCs. Similarly, MDMSCs have recently attracted much interest as osteoproductive cells [59].

Osteoinduction refers to the stimulation of osteogenesis by growth factors that draw the osteogenic cells to the location of a bone defect. Recent isolation of such factors as TGF-*β*3, vascular endothelial growth factor, BMP 2, BMP 4, and BMP 7, has led to their employment in the augmentation, acceleration, replacement, or repair of the bone [60]. Some studies have revealed the potential of recombinant human BMP 2, BMP 3, BMP 4, and BMP 7 fixation of prosthetic implants and in the healing of segmental bone defects [60]. These phenomena are underpinned by the ability of BMP to regulate chemotaxis, mitosis, and differentiation of some cells [60]. To effectively stimulate bone formation, an adequate dose of BMP should be used over a specified period of time [60]. Developments in gene therapy may permit the delivery of genes encoding specific growth factors through engineered MDMSCs or viral vectors, for example, adenoviruses, retroviruses, lentiviruses, and recombinant adeno-associated viral vectors [4, 6, 49, 61].

Osteoconduction depends on the assimilation of a bone cell-bearing structure into a recipient site [60]. In other words, the graft functions as a scaffold that will be ultimately replaced by new tissue and remodeled. The most frequently used bone grafts are frozen allografts, cements, ceramics, and hydroxyapatite, which are typically used without cells [60]. Another hallmark of successful bone tissue engineering is mechanical stimulation, which appears to be vital for the successful generation and differentiation of bone cells and development of bone minerals and matrix configuration [4]. One study [62] compared the effect of core decompression and placement of an autologous bone marrow aspirate into the core decompression track versus core decompression alone as a treatment of osteonecrosis of the femoral head. The aspirate contained elevated numbers of pluripotent MSCs, which signifies that the localization of autologous MSCs in the core decompression track improved the survivorship of femoral heads and reduced the requirement for hip arthroplasty when employed at early stages (precollapse stage). This treatment tactic was sustained on the principle that multipotent MSCs in the bone marrow aspirate would repopulate the trabeculae of the necrotic zone within the femoral head, enhancing regeneration and remodeling of the necrotic bone [62].

Other studies have explored the role of MSCs in lengthening osteotomies [57]. In one study, MSCs were cultivated ex vivo and were used in combination with PRP, leading to accelerated bone healing [57]. Authors have also implanted autologous bone marrow, via percutaneous injection, into aneurismal bone cysts. The healing rates exceeded 80% [57].

Bone formation is relevant to the field of regenerative medicine, especially for the prevention of tissue overgrowth, such as that seen in heterotopic ossification of tendons, ligaments, and muscles [63]. The development of approaches for the control or inhibition of bone formation is indispensable for the prevention of heterotopic ossification. An example of a method of impeding bone formation is the use of the BMP antagonist Noggin, which inhibits bone formation in a dose-dependent manner in mice [63]. However, the therapeutic potential of Noggin in humans remains unresolved [63–65].

## 7. SCs and Nerve Regeneration

Recovery from peripheral nerve injury is generally poor because of the low regeneration rate of axons, and intra- and extraneural scar tissue development [66]. Schwann cells (the key glial cells of the peripheral nervous system) have emerged as key players in peripheral nerve regeneration [66]. SCs are likely to augment the quantity of Schwann cells and extend their ability to maintain nerve regeneration [66]. Currently, the gold standard of bridging material in nerve gaps is the use of autologous nerve grafts. At the same time, their use is associated with high morbidity of harvesting another functioning nerve [66]. Acellular nerve allografts are also frequently employed, although their usefulness is restricted to small distances because of the absence of cellular components and extracellular matrix to assist axonal directional growth [66, 67]. Thus, the inclusion of SCs, growth factors, and extracellular matrix to conduits and allografts may improve their performance by creating a favorable environment for axonal regeneration [66, 67]. In addition, SCs can be transplanted into denervated muscles, avoiding the predictable sequelae of denervation, by the prevention of atrophy, and by leaving the tissue more reinnervation-receptive over extended periods [66, 67].

Nerve SCs can differentiate into neurons and glial cells, most of which are found in the subventricular and subgranular zones of the mammalian adult brain [66]. Recently, neural crest SCs have been isolated from the fetal sciatic nerve of a rat [66], but the presence of those cells in the peripheral nerves of the adult human is not known. Promising results have been obtained following nerve SC implantation into peripheral nerve injuries—not just in acute injuries but also in chronically denervated lesions [66].

BMDMSCs possess the ability to differentiate into nonmesodermal lineages, such as astrocytes, neurons, and Schwann cells [66]. The inclusion of BMDMSCs in conduits and acellular grafts has better outcomes than the ones with cell-depleted grafts [66]. A number of studies showed that ADMSCs possess a bigger SC fraction and superior proliferation and differentiation potential than BMDMSCs [66]. The expression of numerous neuronal and Schwann cell markers on ADMSCs, for example, myelin protein zero, myelin basic protein, and peripheral myelin protein 22, suggests that they may possess a myelin-generating capacity [66, 67]. Further, the effect of ADMSC-filled conduits on nerve regeneration was more favorable than that of cell-deplete grafts [66].

Adipose cell transplantation without a conduit constitutes an alternative approach to the reconstruction of peripheral nerves and has shown some benefit in cases of traumatic brain injury when they are administered systemically [66, 68]. Another study demonstrated that intravenously administered ADMSCs might be used to treat neuropathic pain in rats [69]. The advantage of ADMSCs over BMDMSCs lies in the fact that they are easier to harvest and with a high cellular yield (~0.25–0.375 × 10^6^ cells per mL of liquid fat) [70, 71].

An additional relevant source of MSCs comprises fetal tissues, such as the amniotic fluid and membrane, umbilical cord cells and blood, and Wharton's jelly. Cells harvested from these tissues can differentiate into neural phenotypes [66]. Umbilical cord-derived MSCs and amniotic fluid-derived SCs have been implanted into rodent sciatic nerve defect models and postcrush and transection injury models with promising results [66].

Skin-derived precursors comprise a readily available source of adult multipotent cells. Notably, these cells resemble embryonic neural crest cells [66, 72]. The outcomes of use of undifferentiated and differentiated skin-derived precursors in nerve regeneration are encouraging [66, 72]. In rodent models, skin-derived precursors maintained their ability to differentiate and retain viability upon transplantation, and had the capacity to myelinate axons [72]. In mice, hair follicle SCs express neural markers, for example, Nestin, Nanog, and Oct4, and possess the capacity to differentiate into glial cells and neurons [66, 73–75].

Few studies have analyzed the employment of undifferentiated hair follicle SCs in murine models of sciatic and tibial transection and crush injuries with improved functional outcomes in recipient nerves [66, 73–76]. In addition, differentiated hair follicle SCs placed into acellular xenografts were able to further differentiate for extended periods of time, leading to more regenerated axons with increased myelination as compared to cell-free xenografts [66, 76, 77].

## 8. MSCs in Tendon and Ligament Repair

Tendon and ligament lesions are frequently encountered in orthopedic practice. Once these tissues are injured, they heal by forming a tissue of poorer quality than the native tissue [78–80]. Multiple biological grafts have been used to reconstruct tendons and ligaments, for example, autografts, allografts, and biomaterials, but have been plagued by issues with donor site morbidity, shortage, tissue rejection, and an increased risk of infectious disease transmission [80]. Some studies have examined the use of MSCs for the enhancement of tendon defect repair in animal models. One researcher [4] found that when MSCs are seeded into a defect of an Achilles tendon of a rabbit, the repair encompasses a considerably bigger area and the collagen fibers seemed better aligned than those in control animals. MSCs were also used with collagen composites implanted in patellar tendons of the animals [4]. Healing of these defects was compared in the absence and presence of MSCs, and notably, higher maximum stress and moduli were observed in the MSC group 12 and 26 weeks after the procedure. Other scaffolds, for example, poly(lactide-co-glycolide), loaded with MSCs were also used in the rabbit model to regenerate and repair the Achilles tendon [4]. In another study, human ESCs were used to fix the patellar tendon in rats, leading to the regeneration of tendon tissue in vitro and in vivo [80]. Increased expression of tendon-specific genes and superior structural and mechanical characteristics of the repair sites was noticed in those rats treated with human ESCs [80]. BMDMSCs were also transplanted to various injured tendon sites, resulting in enhanced tissue repair [78]. BMDMSCs in a fibrin glue vehicle were injected into rat patellar tendon defects, resulting in enhanced mature tissue formation and more ordered cell structure than controls [78]. Other authors used ADMSCs and showed that these cells are similar to tendon sheath fibroblasts [80]. Nixon et al. [79, 80] demonstrated that when collagenase-induced tendinitis in the superficial digital flexor tendons in horse forelimbs was managed with ADMSC injections, this resulted in improved tendon organization. In a recent study, Lee et al. [81] isolated multipotent MSCs from a rat tendon, expanded the cells in culture, and exposed to a connective-tissue growth factor. Subsequently, these cells were transplanted with fibrin gel into a transected patellar tendon of a rat; the animals were also treated by a direct injection of the connective-tissue growth factor. The authors observed restoration of the tendon—with correctly aligned collagen fibers and regular tensile strength—after 2 weeks [81, 82].

ACL injuries are extremely frequent and healing of the tendon graft-bone interface is critical to the success of ACL surgical reconstruction [83, 84]. This interface is complicated and composed of four different zones and tissues: ligament substance, unmineralized fibrocartilage, mineralized fibrocartilage, and bone [83, 84]. This anatomy cannot be restored within the initial 6 months postinjury by conventional free tendon transfers [84]; thus, studies have evaluated the utilization of osteoinductive growth factors, for example, TGF, BMP, fibroblast growth factor, and G-CSF, and biomaterials to increase healing of the tendon-bone interface [83, 84]. Atesok et al. [83] demonstrated that BMP 2 can enhance tendon healing in a bone tunnel. Other authors [84] observed that applying these cells to tendon grafts of rabbits resulted in a zone of fibrocartilage, produced at the intersection, which very much features that of a normal ACL. Besides the restoration of the bone-tendon interface, these enhanced grafts have better biomechanical properties [4]. In another study, a tendon-derived SC sheet was wrapped around the tendon graft in a rat model, leading to initial graft healing 12 weeks after ACL reconstruction [31].

## 9. MSC Applications in the Intervertebral Disc, Spine, and Spinal Cord Injuries

Intervertebral disc degeneration is a leading cause of back pain. Many patients are treated with medical interventions, with recovery observed in ~90% of patients [4]. Surgical choices for discogenic back pain are finite and frequently invasive. One of the most frequently performed procedures is discectomy, with or without fusion, although disc replacement has recently received some interest [4]. Nevertheless, no long-term clinical trials assessing the efficacy of the latter procedure have been completed [4, 85].

Some researchers have examined other alternatives, such as transplantation of mature autologous disc cells, chondrocytes, or SCs to the intervertebral disc [85]. Cell transplantation can augment proteoglycan synthesis, decelerate the course of disc degeneration, and stimulate disc regeneration [85]. Transplantation of chondrocytes or autologous disc cells from the costal cartilage was possible and slowed disc degeneration in animal models [4]. In order to explore the viability of exogenous cell delivery, preservation, and survival in the disc, Crevensten et al. [85] injected MSCs into the coccygeal discs of rats by means of 15% hyaluronan gel as a vehicle. On days 7 and 14 postinjection, the SCs were still localized inside the disc, although the number of cells was notably reduced. By day 28, the cell number increased to the initially injected quantity and showed 100% viability. Introduction of genes for the synthesis of growth factors, for example, TGF-*β*1, into disc cells by gene transduction with adenoviral vectors seems to be a promising therapeutic option, albeit very expensive [4].

Currently, the use of autologous bone is considered the best approach to accomplish spinal fusion. However, the rate of nonunion can vary from 5% to 35% [4]. It has been shown that the use of BMPs may result in a rate of fusion similar to or higher than the use of autologous bone. A hybrid graft composed of cultured MSCs with a ceramic scaffold was analyzed; compared with a porous ceramic scaffold alone, the rate of the formation of solid fusion was significantly superior with cultured cells loaded into the porous ceramic scaffold [4, 86].

SC therapy for spinal cord injuries is thought to have significant potential as a result of pluripotent cells to differentiate into neural cells and form neural tissue [87]. Repair or regeneration of the spinal cord is incredibly complicated, as it requires restoring and enhancing the spinal reflex arcs and reconnecting axons. Moreover, gliosis can obstruct axonal outgrowth [4]. Lee et al. [4] showed that following a spinal cord lesion, MSCs isolated from a cultured mononuclear layer of bone marrow can actually remyelinate spinal cord axons following direct injection to the lesion. It is thought that SCs alone may not be sufficient to enhance the function of a damaged spinal cord [87].

BMDMSCs also constitute a promising therapy for spinal cord injuries, but the effect of BMDMSC-only transplantation on spinal cord injuries remains unknown [87]. Minocycline is a second-generation semisynthetic tetracycline with antibacterial properties but it also exerts a significant anti-inflammatory effect. Chen et al. [87] demonstrated that BMDMSCs mixed with minocycline improve spinal cord injury in a rat model, which may represent a promising approach for neuroprotection following spinal cord injury.

## 10. MSCs in Pediatric Orthopedics

Osteogenesis imperfecta (OI) is a genetic disorder characterized by irregularities in type I collagen [4]. OI therapies preferably should be directed to enhancing the structural integrity of collagen, so bone strength can be achieved. The bone marrow possesses cells that can engraft and mature into functional osteoblasts after transplantation as seen in preclinical studies [4]. Because collagen is secreted, a small level of osteoblast engraftment might be helpful to patients with OI [4]. For instance, infused BMDMSCs from a disease-free mouse into OI recipient mice were detected in multiple organs of the receiver mice, including the spleen, bone, lung, and cartilage several months after transplantation [4]. The cells that migrated to the bones differentiated into osteocytes and synthesized standard amounts of collagen type I, partially restoring the OI phenotype. Thus, a possible therapy may include the ex vivo genetic modification of an individual's own bone marrow, followed by a transplantation into the individual, restoring normal collagen levels [4].

Muscular dystrophy is a diverse group of neuromuscular disorders that result in progressive muscle weakness, muscle atrophy, paralysis, and death in severe cases [4]. The current management of some muscular dystrophies involves pharmacological suppression of the immune and inflammatory responses [4]. Unfortunately, beneficial effects of these therapies are only modest and temporary [4]. Significantly, the use of SCs may comprise a solution to treat these pathologies. Wakitani et al. [88] found that, under certain circumstances, in vitro BMDMSCs differentiate into contractile myotubes. Gussoni et al. [89] showed that in an immunodeficient mouse model, marrow-derived cells travel to areas of induced muscle degeneration, where they can experience myogenic differentiation and contribute to the regeneration of injured muscle fibers. Bone marrow or muscle-derived SCs appear to provide a means for systemic, rather than local repair of the muscle, as a result of the distribution of the cells in the vascular system [4].

Injuries of the physis often result in the development of bone bridges among the epiphysis and the metaphysis, and 25–35% of these injuries lead to shortening or angular defect [4, 90]. Stapling, epiphysiodesis, or osteotomies can correct issues in older children; however, treatment is more difficult in younger patients [90]. Other potential therapies have achieved varying degrees of success, such as the removal of the bone bridge or the use of polymeric silicone, fat, or muscle as an interpositional object, but they only serve to avoid the reformation of the bone bridges [90]. In recent times, cultured chondrocytes relocated into physeal defects were shown to correct growth arrest in animal models. Tobita et al. [90] observed that transplantation of autologous chondrocyte cultures in an atelocollagen gel decreased the length incongruity and angular deformity of a leg with an injured physis in a rabbit model. Also, the placement of MSCs into growth-plate defects in rabbits led to an important decrease of the growth arrest of the tibia as shown by Chen et al. [91] In another study in rabbits [4], MSCs were embedded in 10% gelatin in Gelfoam with TGF-*β*3 and transferred into growth-plate defects reduced angular deformity.

Autologous bone marrow transplantation has also been used in other pediatric skeletal diseases, such as osteopetrosis and infantile hypophosphatasia, with promising results [92, 93].

## 11. The Applicability of MSCs in Rotator Cuff Repair

Between 30% and 94% of rotator cuff repairs result in failure, perhaps because the highly specialized fibrocartilaginous transition area connecting the rotator cuff and the bone fails to regenerate following repair [94]. The tissue that is formed after the surgery is a fibrovascular scar tissue, and its mechanical properties are relatively poor [95]. Thus, new materials and surgical techniques have been refined in an effort to augment the strength of the regenerated tissue and replicate the anatomical footprint of the rotator cuff [94–97].

Gulotta and colleagues [98] have investigated the use of MSCs in a fibrin carrier positioned at the tendon-bone interface at the time of rotator cuff repair in a rat model. Engineering of the MSCs to express gene *MT1-MMP*, normally upregulated in the embryo, at the tendon-bone interface appear to increase fibrocartilage deposition at the placement site and improve force after a 4-week follow-up period. Improved orientation of the fibrocartilage fibers was also observed [98].

In addition, the use of BMDMSCs transduced with the transcription factor scleraxis improved tissue biomechanics at 2 weeks and enhanced biomechanics and histology 4 weeks following the establishment of a rotator cuff tear in rats [56]. Moreover, the application of a hydrogel containing periosteum-derived MSCs, polyethylene glycol diacrylate, and BMP 2, at the bone-tendon interface led to histological and biomechanical improvements at 4- and 8-week follow-ups [99]. Further, Kida et al. [100] performed drilling to the greater tuberosity to stimulate the liberation of bone marrow and permit the cells to travel into the suture zone, similar to the microfracture technique in cartilage lesions. At 4 and 8 weeks, SC migration improved maximum load to failure [100]. The use of ADMSCs, MDMSCs, and tenocyte-derived SCs has as well been analyzed in a rotator cuff model with promising results [101–103]. Furthermore, suturing a periosteal flap on top of the torn end of the infraspinatus tendon enhances tendon-to-bone healing in a rabbit rotator cuff model [104].

The only published application of SCs in rotator cuff tears in humans was reported by Gomes et al. [105]. In this study, the authors repaired complete rotator cuff tears in 14 patients using a transosseous approach, enhancing the suture with mononuclear SCs obtained from a bone marrow aspirate from the iliac crest. Based on clinical and magnetic resonance imaging assessment, all 12 tears healed 12 months after the procedure [105].

The effect of PRP application on rotator cuff repair is controversial owing to variable results. For instance, some reports observed clinical improvement in massive tears [106, 107], with accelerated healing and a reduction of postoperative pain in patients with nonmassive rotator cuff tears [108]. Meanwhile, others were unable to find differences in tear healing rates after application of PRP as compared with controls [109, 110]. However, it should be noted that many factors may explain these heterogeneous results, such as the variations in reagent preparation, technique and timing of application, and concentration [111].

## 12. Regulatory Aspects on the Use of Stem Cells

Over the past few years, there has been a lot of excitement regarding stem cell therapies for many diseases beyond the well-established use in hematology [112]. Safety and efficacy are the main aspects to be proven derived from well-controlled clinical trials [112]. In 1962, the Federal Food, Drug, and Cosmetic Act of the United States of America was amended and for the first time the Food and Drug Administration (FDA) demanded phased product development later to be tested in randomized controlled trials [112]. The second breakthrough regarding legislation in the United States was the 21st Century Cures Act of 2016 (Public Law 114-255) that created the designation of “Regenerative Advanced Therapy” in order to expedite their regulatory processing [113]. Despite this major breakthrough, as far as we know, the FDA only has approved stem cell treatments derived from Bone Marrow Aspirate Concentrate (BMAC) but the agency is facing many legal loopholes to fully control autologous stem cell treatments [113]. In Mexico, our group helped in creating the first public bill NOM-260-SSA1-2017 for regulatory laws in the use of stem cell therapies; this public bill is still under public scrutiny, but we aim by the end of 2017 for it to be passed.

## 13. Conclusions

The application of MSCs in medicine is relatively new, but exciting possibilities for the use of these cells in various diseases have already been demonstrated. In the field of orthopedic surgery, experiments involving animals and human subjects indicate that MSCs may be used effectively to treat some diseases and regeneration of some tissues. MSCs can be isolated from several tissues. For tissue engineering and regenerative medicine purposes, they can be obtained from the peripheral blood after mobilization from the blood marrow with specific pharmacologic agents, thus eliminating the requirement of a bone marrow aspirate. These cells can also be acquired during a surgical procedure and can be cryopreserved for future use. Further, MSCs exert anti-inflammatory and immunomodulatory effects and can differentiate into mesenchymal and nonmesenchymal cell types. Nevertheless, the use of MSCs still poses challenges that must be overcome. First, the characterization, differentiation, and expansion procedures used to prepare these cells must be standardized for homogeneous results. Second, tissues have a three-dimensional structure, which demands further research to identify the ideal scaffold for each application based on the tissue's structural, biochemical, and biomechanical properties. Third, neotissues should be functional to replace other injured or diseased tissues. Fourth, these neotissues should be immunologically compatible and not prone to malignancy over time. Last, treatments using MSCs must be cost effective and widely available to everyone who needs them. It is important to recognize that, instead of being a distant goal in the undefined future, the use of MSCs already comprises a realistic option for treating several musculoskeletal diseases. Thus, continued research with animals and human subjects is mandatory to evaluate the safety, efficacy, and applicability of MSCs in orthopedics and in other medical specialties. In orthopedic surgery, the use of MSCs will likely revolutionize the way physicians treat their patients.

## References

[1] Alwattar B. J., Schwarzkopf R., Kirsch T. (2011). Stem cells in orthopaedics and fracture healing. *Bulletin of the NYU Hospital for Joint Diseases*.

[2] Stoddart M. J. (2015). Mesenchymal stem cells as a source of repair cytokines: mesenchymal stem cells as the conductor. *Journal of the American Academy of Orthopaedic Surgeons*.

[3] Stoltz J. F., de Isla N., Li Y. P. (2015). Stem cells and regenerative medicine: myth or reality of the 21st century. *Stem Cells International*.

[4] Lee E. H., Hui J. H. P. (2006). The potential of stem cells in orthopaedic surgery. *Journal of Bone and Joint Surgery*.

[5] Gómez-Barrena E., Solá C. A., Bunu C. P. (2014). Regulatory authorities and orthopaedic clinical trials on expanded mesenchymal stem cells. *International Orthopaedics*.

[6] Corsi K. A., Schwartz E. M., Mooney D. J., Huard J. (2007). Regenerative medicine in orthopaedic surgery. *Journal of Orthopaedic Research*.

[7] Bobis S., Jarocha D., Majka M. (2006). Mesenchymal stem cells: characteristics and clinical applications. *Folia Histochemica et Cytobiologica*.

[8] Steinert A. F., Kunz M., Prager P. (2015). Characterization of bursa subacromialis-derived mesenchymal stem cells. *Stem Cell Research and Therapy*.

[9] Caplan A. I. (2015). Adult mesenchymal stem cells: when, where, and how. *Stem Cells International*.

[10] Mazaheri M., Eslahi N., Ordikhani F., Tamjid E., Simchi A. (2015). Nanomedicine applications in orthopaedic medicine: state of the art. *International Journal of Nanomedicine*.

[11] Geris L. (2014). Regenerative orthopaedics: in vitro, in vivo…in silico. *International Orthopaedics*.

[12] Patterson T. E. (2008). Cellular strategies for enhancement of fracture repair. *The Journal of Bone & Joint Surgery*.

[13] Marcucio R. S., Nauth A., Giannoudis P. V. (2015). Stem cell therapies in orthopaedic trauma. *Journal of Orthopaedic Trauma*.

[14] Barry F., Murphy M. (2013). Mesenchymal stem cells in joint disease and repair. *Nature Reviews Rheumatology*.

[15] Landa-Solís C., Granados-Montiel J., Olivos-Meza A. (2016). Cryopreserved CD90+ cells obtained from mobilized peripheral blood in sheep: a new source of mesenchymal stem cells for preclinical applications. *Cell and Tissue Banking*.

[16] Dominici M., Le Blanc K., Mueller I. (2006). Minimal criteria for defining multipotent mesenchymal stromal cells. The International Society for Cellular Therapy position statement. *Cytotherapy*.

[17] Hernigou P., Homma Y., Flouzat-Lachaniette C. H., Poignard A., Chevallier N., Rouard H. (2013). Cancer risk is not increased in patients treated for orthopaedic diseases with autologous bone marrow cell concentrate. *The Journal of Bone & Joint Surgery*.

[18] Colnot C., Zhang X., Knothe Tate M. L. (2012). Current insights on the regenerative potential of the periosteum: molecular, cellular, and endogenous engineering approaches. *Journal of Orthopaedic Research*.

[19] Watanabe Y., Harada N., Sato K., Abe S., Yamanaka K., Matushita T. (2016). Stem cell therapy: is there a future for reconstruction of large bone defects?. *Injury*.

[20] Bernhardsson M., Sandberg O., Aspenberg P. (2015). Experimental models for cancellous bone healing in the rat. *Acta Orthopaedica*.

[21] Hernigou P., Poignard A., Beaujean F., Rouard H. (2005). Percutaneous autologous bone-marrow grafting for nonunions. Influence of the number and concentration of progenitor cells. *Journal of Bone and Joint Surgery*.

[22] Cui L., Yin S., Liu W., Li N., Zhang W., Cao Y. (2007). Expanded adipose-derived stem cells suppress mixed lymphocyte reaction by secretion of prostaglandin E2. *Tissue Engineering*.

[23] Tseng S. S., Lee M. A., Reddi A. H. (2008). Nonunions and potential of stem cells in fracture-healing. *The Journal of Bone & Joint Surgery*.

[24] Peçanha R., De Lima L., Ribeiro M. B. (2012). Adipose-derived stem-cell treatment of skeletal muscle injury. *The Journal of Bone & Joint Surgery*.

[25] Qu-Petersen Z., Deasy B., Jankowski R. (2002). Identification of a novel population of muscle stem cells in mice: potential for muscle regeneration. *Journal of Cell Biology*.

[26] Gates C. B., Karthikeyan T., Fu F., Huard J. (2008). Regenerative medicine for the musculoskeletal system based on muscle-derived stem cells. *The Journal of the American Academy of Orthopaedic Surgeons*.

[27] Grässel S., Lorenz J. (2014). Tissue-engineering strategies to repair chondral and osteochondral tissue in osteoarthritis: use of mesenchymal stem cells. *Current Rheumatology Reports*.

[28] Mujeeb A., Ge Z. (2014). On the horizon from the ORS: biomaterials for cartilage regeneration. *The Journal of the American Academy of Orthopaedic Surgeons*.

[29] Araki S., Imai S., Ishigaki H. (2015). Improved quality of cartilage repair by bone marrow mesenchymal stem cells for treatment of an osteochondral defect in a cynomolgus macaque model. *Acta Orthopaedica*.

[30] Zhou C., Zheng H., Seol D., Yu Y., Martin J. A. (2014). Gene expression profiles reveal that chondrogenic progenitor cells and synovial cells are closely related. *Journal of Orthopaedic Research*.

[31] Rodeo S. A., Lebaschi A., Carballo C. (2015). What’s new in orthopaedic research. *The Journal of Bone & Joint Surgery*.

[32] Atesok K., Doral M. N., Bilge O., Sekiya I. (2013). On the horizon from the ORS synovial stem cells in musculoskeletal. *The Journal of the American Academy of Orthopaedic Surgeons*.

[33] Koga H., Muneta T., Ju Y. J. (2007). Synovial stem cells are regionally specified according to local microenvironments after implantation for cartilage regeneration. *Stem Cells*.

[34] Nakamura T., Sekiya I., Muneta T. (2012). Arthroscopic, histological and MRI analyses of cartilage repair after a minimally invasive method of transplantation of allogeneic synovial mesenchymal stromal cells into cartilage defects in pigs. *Cytotherapy*.

[35] Goodrich L. R., Chen A. C., Werpy N. M. (2016). Addition of mesenchymal stem cells to autologous platelet-enhanced fibrin scaffolds in chondral defects. *The Journal of Bone & Joint Surgery*.

[36] Frisbie D. D., McCarthy H. E., Archer C. W., Barrett M. F., McIlwraith C. W. (2015). Evaluation of articular cartilage progenitor cells for the repair of articular defects in an equine model. *The Journal of Bone & Joint Surgery*.

[37] Matsukura Y., Muneta T., Tsuji K., Koga H., Sekiya I. (2014). Mesenchymal stem cells in synovial fluid increase after meniscus injury. *Clinical Orthopaedics and Related Research*.

[38] Broderick J. M., Kelly D. J., Mulhall K. J. (2014). Optimizing stem cell engineering for orthopaedic applications. *The Journal of the American Academy of Orthopaedic Surgeons*.

[39] Sun L., Reagan M. R., Kaplan D. L. (2010). Role of cartilage forming cells in regenerative medicine for cartilage repair. *Journal of Orthopedic Research and Reviews*.

[40] Sakata R., McNary S. M., Miyatake K. (2015). Stimulation of the superficial zone protein and lubrication in the articular cartilage by human platelet-rich plasma. *The American Journal of Sports Medicine*.

[41] Sierra R., Wyles C., Houdek M., Behfar A. (2015). Mesenchymal stem cell therapy for osteoarthritis: current perspectives. *Stem Cells Cloning*.

[42] Tuan R. S., Chen A. F., Klatt B. A. (2013). Cartilage regeneration. *Journal of the American Academy of Orthopaedic Surgeons*.

[43] Murphy J. M., Fink D. J., Hunziker E. B., Barry F. P. (2003). Stem cell therapy in a caprine model of osteoarthritis. *Arthritis Rheumatology*.

[44] Horie M., Choi H., Lee R. H. (2012). Intra-articular injection of human mesenchymal stem cells (MSCs) promote rat meniscal regeneration by being activated to express Indian hedgehog that enhances expression of type II collagen. *Osteoarthritis and Cartilage*.

[45] Lee C. H., Cook J. L., Mendelson A., Moioli E. K., Yao H., Mao J. J. (2010). Regeneration of the articular surface of the rabbit synovial joint by cell homing: a proof of concept study. *Lancet*.

[46] Marini J. C., Forlino A. (2012). Replenishing cartilage from endogenous stem cells. *New England Journal of Medicine*.

[47] Frisbie D. D., Kisiday J. D., Kawcak C. E. (2009). Evaluation of adipose-derived stromal vascular fraction or bone marrow-derived mesenchymal stem cells for treatment of osteoarthritis. *Journal of Orthopaedic Research*.

[48] Johnson K., Zhu S., Tremblay M. S. (2012). A stem cell-based approach to cartilage repair. *Science*.

[49] Orth P., Rey-Rico A., Venkatesan J. K., Madry H., Cucchiarini M. (2014). Current perspectives in stem cell research for knee cartilage repair. *Stem Cells Cloning*.

[50] Sillat T., Barreto G., Clarijs P. (2013). Toll-like receptors in human chondrocytes and osteoarthritic cartilage. *Acta Orthopaedica*.

[51] Zellner J., Mueller M., Berner A. (2010). Role of mesenchymal stem cells in tissue engineering of meniscus. *Journal of Biomedical Materials Research A*.

[52] Dutton A. Q., Choong P. F., Goh J., Lee E. H., Hui J. H. (2010). Enhancement of meniscal repair in the avascular zone using mesenchymal stem cells in a porcine model. *The Journal of Bone & Joint Surgery*.

[53] Kraeutler M. J., Mitchell J. J., Chahla J., McCarty E. C., Pascual-Garrido C. (2017). Intra-articular implantation of mesenchymal stem cells, part 1: a review of the literature for prevention of postmeniscectomy osteoarthritis. *Orthopaedic Journal of Sports Medicine*.

[54] Kraeutler M. J., Mitchell J. J., Chahla J., McCarty E. C., Pascual-Garrido C. (2017). Intra-articular implantation of mesenchymal stem cells, part 2: a review of the literature for meniscal regeneration. *Orthopaedic Journal of Sports Medicine*.

[55] Horie M., Driscoll M. D., Sampson H. W. (2012). Implantation of allogenic synovial stem cells promotes meniscal regeneration in a rabbit meniscal defect model. *The Journal of Bone & Joint Surgery*.

[56] Anz A. W., Hackel J. G., Nilssen E. C., Andrews J. R. (2014). Application of biologics in the treatment of the rotator cuff, meniscus, cartilage, and osteoarthritis. *The Journal of the American Academy of Orthopaedic Surgeons*.

[57] Jäger M., Hernigou P., Zilkens C. (2010). Cell therapy in bone healing disorders. *Orthopedic Reviews*.

[58] Cuomo A. V., Virk M., Petrigliano F., Morgan E. F., Lieberman J. R. (2009). Mesenchymal stem cell concentration and bone repair: potential pitfalls from bench to bedside. *The Journal of Bone & Joint Surgery*.

[59] Pipino C., Pandolfi A. (2015). Osteogenic differentiation of amniotic fluid mesenchymal stromal cells and their bone regeneration potential. *World Journal of Stem Cells*.

[60] De Long W. G., Einhorn T. A., Koval K. (2007). Bone grafts and bone graft substitutes in orthopaedic trauma surgery. A critical analysis. *World Journal of Stem Cells*.

[61] Musgrave D. S., Fu F. H., Huard J. (2002). Gene therapy and tissue engineering in orthopaedic surgery. *The Journal of the American Academy of Orthopaedic Surgeons*.

[62] Papakostidis C., Tosounidis T. H., Jones E., Giannoudis P. V. (2016). The role of “cell therapy” in osteonecrosis of the femoral head. *Acta Orthopaedica*.

[63] Hannallah D., Peng H., Young B., Usas A., Gearhart B., Huard J. (2004). Retroviral delivery of Noggin inhibits the formation of heterotopic ossification induced by BMP-4, demineralized bone matrix, and trauma in an animal model. *The Journal of Bone & Joint Surgery*.

[64] Black C. R. M., Goriainov V., Gibbs D., Kanczler J., Tare R. S., Oreffo R. O. (2015). Bone tissue engineering. *Current Molecular Biology Reports*.

[65] Harris M. B. (2008). Perspectives on modern orthopaedics recent developments in the biology of fracture repair. *The Journal of the American Academy of Orthopaedic Surgeons*.

[66] Fairbairn N. G., Meppelink A. M., Ng-Glazier J., Randolph M. A., Winograd J. M. (2015). Augmenting peripheral nerve regeneration using stem cells: a review of current opinion. *World Journal of Stem Cells*.

[67] Johnson P. J., Wood M. D., Moore A. M., Mackinnon S. E. (2013). Tissue engineered constructs for peripheral nerve surgery. *European Surgery*.

[68] Tajiri N., Acosta S. A., Shahaduzzaman M. (2014). Intravenous transplants of human adipose derived stem cell protect the brain from traumatic brain injury-induced neurodegeneration and motor and cognitive impairments: cell graft biodistribution and soluble factors in young and aged rats. *Journal of Neuroscience*.

[69] Sacerdote P., Niada S., Franchi S. (2013). Systemic administration of human adipose-derived stem cells reverts nociceptive hypersensitivity in an experimental model of neuropathy. *Stem Cells Development*.

[70] Gimble J. M., Bunnell B. A., Frazier T. (2013). Adipose-derived stromal/stem cells: a primer. *Organogenesis*.

[71] Zack-Williams S. D. L., Butler P. E., Kalaskar D. M. (2015). Current progress in use of adipose derived stem cells in peripheral nerve regeneration. *World Journal of Stem Cells*.

[72] McKenzie I. A., Biernaskie J., Toma J. G., Midha R., Miller F. D. (2006). Skin-derived precursors generate myelinating Schwann cells for the injured and dysmyelinated nervous system. *Journal of Neuroscience*.

[73] Amoh Y., Aki R., Hamada Y. (2012). Nestin-positive hair follicle pluripotent stem cells can promote regeneration of impinged peripheral nerve injury. *Journal of Dermatology*.

[74] Amoh Y., Kanoh M., Niiyama S. (2009). Human hair follicle pluripotent stem (hfPS) cells promote regeneration of peripheral-nerve injury: an advantageous alternative to ES and iPS cells. *Journal of Cellular Biochemistry*.

[75] Amoh Y., Li L., Campillo R. (2005). Implanted hair follicle stem cells form Schwann cells that support repair of severed peripheral nerves. *Proceedings of the National Academy of Science USA*.

[76] Lin H., Liu F., Zhang C. (2009). Pluripotent hair follicle neural crest stem-cell-derived neurons and Schwann cells functionally repair sciatic nerves in rats. *Molecular Neurobiology*.

[77] Muheremu A., Ao Q. (2015). Past, present, and future of nerve conduits in the treatment of peripheral nerve injury. *BioMedical Research International*.

[78] Okamoto N., Kushida T., Oe K., Umeda M., Ikehara S., Iida H. (2010). Treating Achilles tendon rupture in rats with bone-marrow-cell transplantation therapy. *The Journal of Bone & Joint Surgery*.

[79] Nixon A. J., Dahlgren L. A., Haupt J. L., Yeager A. E., Ward D. L. (2008). Effect of adipose derived nucleated cell fractions on tendon repair in horses with collagenase induced tendinitis. *American Journal of Veterinary Research*.

[80] Hogan M. V., Bagayoko N., James R., Starnes T., Katz A., Chhabra A. B. (2011). Tissue engineering solutions for tendon repair. *Journal of the American Academy of Orthopaedic Surgeons*.

[81] Lee C. H., Lee F. Y., Tarafder S. (2015). Harnessing endogenous stem/progenitor cells for tendon regeneration. *Journal of Clinical Investigation*.

[82] Phimister E. G., Prockop D. J. (2015). Hardly tendentious-repairing like with like. *New England Journal of Medicine*.

[83] Hogan M. C., Kawakami Y., Murawski C. D., Fu F. H. (2015). Tissue engineering of ligaments for reconstructive surgery. *Arthroscopy*.

[84] Atesok K., Fu F. H., Wolf M. R. (2014). Augmentation of tendon-to-bone healing. *The Journal of Bone & Joint Surgery*.

[85] Crevensten G., Walsh A. J., Ananthakrishnan D. (2004). Intervertebral disc cell therapy for regeneration: mesenchymal stem cell implantation in rat intervertebral discs. *Annals of Biomedical Engineering*.

[86] Hsu W. K. (2014). Recombinant human bone morphogenetic protein-2 in spine surgery. *The Journal of Bone & Joint Surgery*.

[87] Chen D., Zeng W., Fu Y., Gao M., Lv G. (2015). Bone marrow mesenchymal stem cells combined with minocycline improve spinal cord injury in a rat model. *International Journal of Clinical and Experimental Pathology*.

[88] Wakitani S., Saito T., Caplan A. (1995). Myogenic cells derived from rat bone marrow mesenchymal stem cells exposed to 5-azacytidine. *Muscle & Nerve*.

[89] Gussoni E., Soneoka Y., Strickland C. D. (1999). Dystrophin expression in the MDX mouse restored by stem cell transplantation. *Nature*.

[90] Tobita M., Ochi M., Uchio Y. (2002). Treatment of growth plate injury with autogenous chondrocytes: a study in rabbits. *Acta Orthopaedica*.

[91] Chen F., Hui J. H., Chan W. K., Lee E. H. (2003). Cultured mesenchymal stem cell transfers in the treatment of partial growth arrest. *Journal of Pediatric Orthopaedics*.

[92] Driessen G. J., Gerritsen E. J., Fischer A. (2003). Long-term outcome of haematopoietic stem cell transplantation in autosomal recessive osteopetrosis: an EBMT report. *Bone Marrow Transplantation*.

[93] Cahill R. A., Wenkert D., Perlman S. A. (2007). Infantile hypophosphatasia: transplantation therapy trial using bone fragments and cultured osteoblasts. *The Journal of Clinical Endocrinology & Metabolism*.

[94] Kim H. M., Caldwell J. M., Buza J. A. (2014). Factors affecting satisfaction and shoulder function in patients with a recurrent rotator cuff tear. *The Journal of Bone & Joint Surgery*.

[95] Bishop J., Klepps S., Lo I. K., Bird J., Gladstone J. N., Flatow E. L. (2006). Cuff integrity after arthroscopic versus open rotator cuff repair: a prospective study. *Journal of Shoulder and Elbow Surgery*.

[96] Tashjian R. Z., Hollins A. M., Kim H. M. (2010). Factors affecting healing rates after arthroscopic double-row rotator cuff repair. *American Journal of Sports Medicine*.

[97] Mora M. V., Ruiz M. A., Díaz J., Barco Laakso R., Cuéllar R., García Arranz M. (2015). Stem cell therapy in the management of shoulder rotator cuff disorders. *World Journal of Stem Cells*.

[98] Gulotta L. V., Kovacevic D., Montgomery S., Ehteshami J. R., Packer J. D., Rodeo S. A. (2010). Stem cells genetically modified with the developmental gene MT1-MMP improve regeneration of the supraspinatus tendon-to-bone insertion site. *American Journal of Sports Medicine*.

[99] Chen C. H., Chang C. H., Wang K. C. (2011). Enhancement of rotator cuff tendon bone healing with injectable periosteum progenitor cells-BMP-2 hydrogel in vivo. *Knee Surgery, Sports Traumatology, Arthroscopy*.

[100] Kida Y., Morihara T., Matsuda K. (2013). Bone marrow-derived cells from the footprint infiltrate into the repaired rotator cuff. *Journal of Shoulder and Elbow Surgery*.

[101] Tornero-Esteban P., Hoyas J. A., Villafuertes E. (2015). Efficacy of supraspinatus tendon repair using mesenchymal stem cells along with a collagen I scaffold. *Journal of Orthopaedic Surgery and Research*.

[102] Oh J. H., Chung S. W., Kim S. H., Chung J. Y., Kim J. Y. (2014). 2013 Neer award: effect of the adipose-derived stem cell for the improvement of fatty degeneration and rotator cuff healing in rabbit model. *Journal of Shoulder and Elbow Surgery*.

[103] Shen W., Chen J., Yin Z. (2012). Allogenous tendon stem/progenitor cells in silk scaffold for functional shoulder repair. *Cell Transplantation*.

[104] Chang C. H., Chen C. H., Su C. Y., Liu H. T., Yu C. M. (2009). Rotator cuff repair with periosteum for enhancing tendon-bone healing: a biomechanical and histological study in rabbits. *Knee Surgery, Sports Traumatology, Arthroscopy*.

[105] Ellera Gomes J. L., da Silva R. C., Silla L. M., Abreu M. R., Pellanda R. (2012). Conventional rotator cuff repair complemented by the aid of mononuclear autologous stem cells. *Knee Surgery, Sports Traumatology, Arthroscopy*.

[106] Sánchez Márquez J. M., Martínez Díez J. M., Barco R., Antuña S. (2011). Functional results after arthroscopic repair of massive rotator cuff tears; influence of the application platelet-rich plasma combined with fibrin. *Revista Española de Cirugía Ortopédica Y Traumatología (English Edition)*.

[107] Ruiz-Moneo P., Molano-Muñoz J., Prieto E., Algorta J. (2013). Plasma rich in growth factors in arthroscopic rotator cuff repair: a randomized, double-blind, controlled clinical trial. *Arthroscopy*.

[108] Randelli P., Arrigoni P., Ragone V., Aliprandi A., Cabitza P. (2011). Platelet rich plasma in arthroscopic rotator cuff repair: a prospective RCT study, 2-year follow-up. *Journal of Shoulder and Elbow Surgery*.

[109] Weber S. C., Kauffman J. I., Parise C., Weber S. J., Katz S. D. (2013). Platelet-rich fibrin matrix in the management of arthroscopic repair of the rotator cuff: a prospective, randomized, double-blinded study. *American Journal of Sports Medicine*.

[110] Bergeson A. G., Tashjian R. Z., Greis P. E., Crim J., Stoddard G. J., Burks R. T. (2012). Effects of platelet-rich fibrin matrix on repair integrity of at-risk rotator cuff tears. *American Journal of Sports Medicine*.

[111] Abtahi A. M., Granger E. K., Tashjian R. Z. (2015). Factors affecting healing after arthroscopic rotator cuff repair. *World Journal of Orthopaedics*.

[112] Marks P. W., Witten C. M., Califf R. M. (2017). Clarifying stem-cell therapy’s benefits and risks. *New England Journal of Medicine*.

[113] Daley G. Q. (2017). Polar extremes in the clinical use of stem cells. *New England Journal of Medicine*.

